# Comparative evaluation of hydrogen peroxide sporicidal efficacy by different standard test methods

**Published:** 2020-04

**Authors:** Simin Sadeghi, Soosan Abdollahi, Parastoo Tarighi, Nasrin Samadi

**Affiliations:** 1 Department of Drug and Food Control, School of Pharmacy, Tehran University of Medical Sciences, Tehran, Iran; 2 Department of Pharmaceutics, Faculty of Pharmacy, Lorestan University of Medical Sciences, Khorramabad, Iran; 3 Department of Medical Biotechnology, School of Allied Medicine, Iran University of Medical Sciences, Tehran, Iran; 4 Pharmaceutical Quality Assurance Research Center, The Institute of Pharmaceutical Sciences (TIPS), Tehran University of Medical Sciences, Tehran, Iran

**Keywords:** Hydrogen peroxide, Sporicidal test, Suspension test, Carrier test, *Bacillus subtilis* spore

## Abstract

**Background and Objectives:**

There are different sporicidal standard tests with various specifications to deal with products that are claimed for sporicidal activity. The aim of this study was to compare the 7% H_2_O_2_ sporicidal efficacy against *Bacillus subtilis* spores using different standard test methods.

**Materials and Methods:**

The 7% H_2_O_2_ sporicidal efficacy against *Bacillus subtilis* spores was determined according to the AOAC MB-15-04 standard of carrier test and two standard suspension tests (BS EN 13704, AFNOR NF 72-230) in both clean and dirty conditions and by using different interfering substances including bovine serum albumin, yeast extract and skimmed milk.

**Results:**

The results of suspension tests with 3 × 10^5^ and 2 × 10^7^ CFU/ml of *B. subtilis* spore concentration demonstrated that the higher spore counts lead to lower efficacy of 7% H_2_O_2_. Also, the sporicidal activity of 7% H_2_O_2_ was reduced in the presence of interfering substances. Bovine serum albumin, yeast, and skimmed milk showed similar interfering effects in suspension test with 3 × 10^5^ CFU/ml. While, in suspension tests with higher initial spore count (2 × 10^7^ CFU/ml) severity of interfering effects were intensified and distinct. Our results indicated that the carrier sporicidal test in comparison with suspension tests required more contact time to kill *B. subtilis* spores.

**Conclusion:**

The results of this study showed that it is reasonable to use interfering substances and inoculated carriers in accordance with actual conditions of product usage in a sporicidal test. Interfering substances may reduce the contact surface between H_2_O_2_ and test spores; therefore, the sporicidal efficacy of H_2_O_2_ was diminished. So applying suspension test in clean condition to verify the claim of sporicidal activity is strongly discouraged.

## INTRODUCTION

The bacterial endospore production occurs in challenging by unfavorable environmental conditions (1). Since the spores are highly resistant to adverse environments such as lack of moisture or essential nutrients, toxic chemicals, radiations and high temperatures, the environmental protection agency (EPA) considers a sporicidal agent as sterilizer (2). A sterilizer is an antimicrobial agent that destroys or eliminates all forms of microbial life in the inanimate environment (3).

Hydrogen peroxide (H_2_O_2_) is one of the sporicidal chemical compounds which is not toxic at residual concentrations. It is used as a sterilizer for processing equipment and packaging materials in aseptic packaging processes (4, 5).

The quantitative disinfectant efficacy tests fall into one of three categories, i.e. suspension tests, carrier tests or surface tests (6). They have common procedures including: contact between test compound and indicating microbial strain for a known contact time, inactivation of the disinfectant compound, recovery and determination of the number of survived target organisms and calculation of reduction factor. In this regard, recovery is not considered as an ability to repair damage, but only to germinate and multiply in a suitable nutrient environment (7).

Suspension tests are the simplest form of quantitative tests and there is good mixing between the tests disinfectant and indicating strain which improves the reproducibility of the test. On the other hand, the carrier test is providing the possibility of using large numbers of carriers per each run to increase the sensitivity of the test. Totally, the transition from suspension test to carrier or surface tests conduct the test condition to actual conditions of product usage.

There are diverse methods to determine the efficacy of sporicidal agents with different specifications. These differences are related to interfering substances, indicating strain, initial spore concentration, data presentation, type of test method (suspension, carrier, surface) and test conditions (clean, dirty). Because of this diversity in the items of sporicidal test methods, their ability to provide an equivalence evaluation is always under discussion. For example, European sporicidal suspension tests are run either in the clean conditions or in the presence of low levels (0.3g/l) of bovine serum albumin or other proteins such as tryptone, mucin, or milk to simulate organic contamination (8–10). Also, the European and U.S sporicidal tests specify different strains of *Bacillus subtilis, Bacillus cereus* and *Clostridium sporogenes* as indicating strains (11).

To determine the effect of different initial inoculum size, presence of interfering substances, and surface adhesion of bacteria on sporicidal activity of high level disinfectants, this paper attempts to compare the results of testing a sporicidal agent (7% H_2_O_2_) against *B. subtilis* spores according to the suspension tests described by BS EN 13704 (9) and AFNOR NF 72-230 (12), and the carrier test of AOAC MB-15-04 (13) in both clean and dirty conditions by using different interfering substances.

## MATERIALS AND METHODS

### Test solutions.

The 7% (v/v) hydrogen peroxide solution was made from 35% H_2_O_2_ stock solution (Merck Co., Germany) prior to each test. Permanganometric titration was run according to the USP 40 in order to determine the concentration of H_2_O_2_ stock solution (14).

The organization for economic co-operation and development (OECD) recommended the 375 ppm hard water as a diluent for test substance (15). The hard water was prepared according to European committee for standardization (CEN)-EN 13727 method (16). The solution A was obtained by dissolving 19.84 g anhydrous magnesium chloride and 46.24 g anhydrous calcium chloride in 1000 ml of de-ionized water and sterilized by autoclaving. The solution B was prepared by dissolving 35.02 g sodium bicarbonate in 1000 ml of water and sterilizing by membrane filtration. To prepare 375 ppm hard water, 6 ml of solution A and 8 ml of solution B were added to de-ionized water and the final volume was brought to 1000 ml. The pH of the hard water was adjusted to 7 ± 0.2 by using 1 M NaOH or 1 M HCl.

### Spore preparation.

Spore suspension of *Bacillus subtilis* ATCC 6633 was prepared according to the AOAC MB-15-04 sporicidal standard test (13). Briefly, an overnight culture of *B. subtilis* that was obtained from Persian Type Culture Collection was used to inoculate the sporulation medium. The sporulation medium consisted of nutrient agar supplemented with 5 μg/ml of MnSO_4_.H_2_O (17). After 21 days of incubation at 36 ± 1 ºC, the *B. subtilis* spores were harvested, washed three times with cold sterile water and stored at 4 ºC. The purity of the spore suspension was checked by using optical microscopy and staining with malachite green. The total viable spore count was determined by serial dilution and pour plating on tryptic soy agar (TSA) medium.

### Neutralization validation.

A neutralization confirmation test was performed according to the USP 40 in order to demonstrate the recovery of a low level of spores (18). For this purpose, three tubes including one test tube (9 ml neutralizer + 1 ml of 7% H_2_O_2_), one viability tube (10 ml of 0.1% peptone), and one peptone control tube (1 ml of 0.1% peptone + 9 ml neutralizer) were considered and inoculated with *B. subtilis* spores to reach the final concentration of about 100 spores/ml. After about 15–20 min, the recovery ratios were determined. Ratio of test tube to viability tube count demonstrated neutralizer efficacy and the ratio of peptone control group to viability group demonstrated neutralizer toxicity.

The letheen broth (lecithin, 0.07%; sodium chloride, 0.5%; beef extract, 0.5%; peptone, 1%; Tween 80, 0.5%) supplemented with 0.1, 0.5, 0.6, 1, 1.5, 2, and 3% (w/v) sodium thiosulfate, the MnO_2_ (0.3. 0.5, and 1%) in letheen broth or distilled water and FeCl_3_ (0.5, and 1%) in distilled water were used as disinfectant neutralizers.

### Suspension test.

This test was performed based on the two standard methods, BS EN 13704 and AFNOR NF 72-230 (9, 12). For dirty condition, 1 ml of interfering substance solution (0.3% bovine serum albumin, 1% yeast extract, or 1% skimmed milk) and for clean condition 1 ml of distilled water were added to the 9 ml of 7% H_2_O_2_ solution that was inoculated with spore suspension. The initial spore concentration was 3 × 10^5^ or 2 × 10^ 7^ CFU/ml, according to the BS EN 13704 or AFNOR NF 72_230 test methods, respectively. After passing the contact time (2–17 min), 1 ml of each test mixture was added to 9 ml of the neutralizer. The viable spore counts were determined by serial dilution of 1 ml of neutralized mixture and pour plating on TSA medium after 3 days at 36 ± 1 ºC. At each set of test, a H_2_O_2_ free tube was used as control.

### Carrier test.

The AOAC sporicidal standard method MB-15-04 was employed as a classical carrier based standard test (13). Each of the stainless steel carrier (8 ± 1 mm OD, 6 ± 1 mm ID, 10 ± 1 mm length) was brushed, sterilized and separately placed in a tube containing 10 ml of spore suspension (1.0 × 10^ 8^ CFU/ml). After 15 minutes, carriers were removed and dried under a laminar flow cabinet. The spore number on each carrier was counted through plate counting. In this regard, 5 carriers were selected randomly. Each of them was sonicated for 5 min ± 30s in a 50 ml conical centrifuge tube containing 10 ml of sterile water followed by 2 min ± 5 s vortexing. Suspensions in the conical tubes were diluted and 100 μl of each dilution was incubated on TSA plates at 36 ± 1 °C and counted after 3 days. The final carrier count should be about 5 × 10^5^ CFU /carrier.

The acid- resistance test of spores was carried out according to the AOAC sporicidal Method MB-15-04 to evaluate the quality of spores which were inoculated on the carriers (13). First, two tubes containing 10 ml of 2.5 N HCl were placed into a 20 ± 1 ºC water bath. Then, four inoculated carriers were transferred into an acid tube, and were transferred individually after 2, 5, 10 and 20 min exposure to a separate tube of fluid thioglycollate medium with 1 M NaOH (modified FTM). The tubes were rotated vigorously for 20 seconds and then the carriers were put to a second tube of modified FTM. An unexposed inoculated carrier had been placed in a separate tube of modified FTM as a control of viability and a tube of modified FTM was used for sterility control. All test and control tubes were incubated at 36 ± 1 ºC for 3 days. The spores should be resistant to 2.5 N HCl for more than 2 min to achieve qualification as acid-resistant test spores.

For carrier test, a set of five inoculated stainless steel carriers was exposed to 10 ml of 7% H_2_O_2_ for each contact time (1–60 min). Then, carriers were transferred into the first subculture tube containing tryptic soy broth (TSB) with 0.5% MnO_2_. After an hour, each of the carriers was placed into the second subculture tube of 10 ml TSB medium. Both neutralizer tubes (without carrier) and recovery medium tubes (with carrier) were incubated 3 days at 36 ± 1 ºC. When turbidity occurred, in any of the primary or secondary subculture tubes, positive result was considered for that carrier set. The results were reported as growth (+) or no growth (0). Suitable controls were incorporated to check for sterility of media, reagents, and carriers.

## RESULTS

### Spore preparation.

**Fig. 1.**
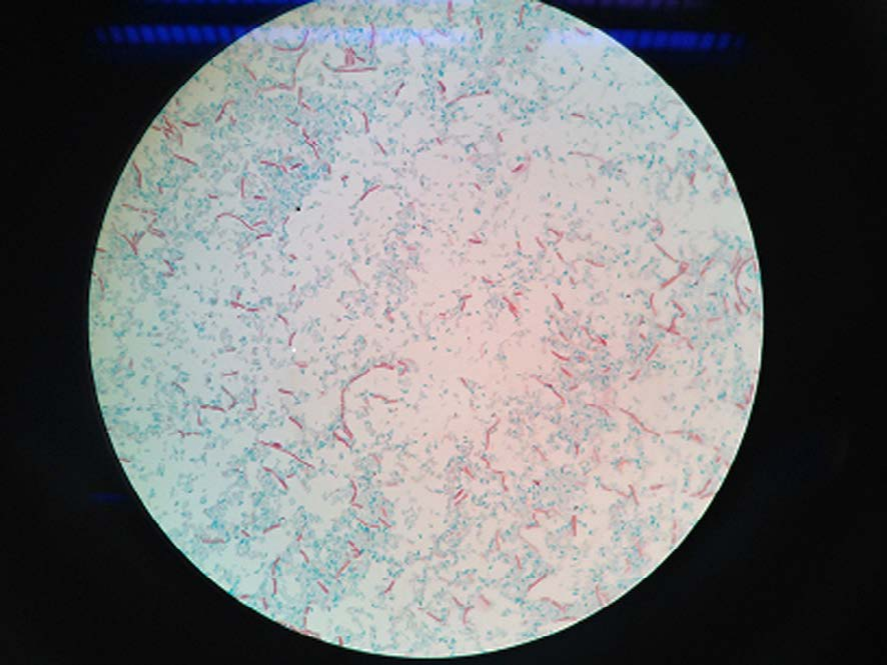
Optical microscopy of *Bacillus subtilis* spores after staining with malachite green

The method of spore purification recommended by sporicidal standards (AOAC MB-15-04, BS EN 13704) generally include successive steps of washing and centrifugation (9, 13). By using AOAC method, after daily examination of culture medium by optical microscopy and staining with malachite green, it was founded that 21-day culture of *B. subtilis* had high spore purity (Fig. 1). Our spore suspension had a concentration of about 1.5 × 10^9^ CFU/ml.

### Neutralization validation.

An effective neutralization method for a chemical biocide is critically important for the accuracy of biocidal assay (19). Therefore, here we attempted to compare three different chemical neutralizers and select the best one due to its acceptable toxicity percentage and neutralizing efficacy.

As shown in Table 1, all sodium thiosulfate dilutions (0.5, 0.6, 1, 1.5, 2 and 3%) in letheen broth and FeCl_3_ in distilled water showed acceptable rate of neutralizer toxicity (≥50%) but no proper neutralizing efficacy. All three dilutions of MnO_2_ (0.3, 0.5, 1) in letheen broth or distilled water passed neutralization validation test (≥50% neutralizer efficacy and toxicity). Therefore, 0.5% (w/v) of MnO_2_ in distilled water and letheen broth were used as neutralizers for suspension test and carrier test, respectively.

**Table 1.** Neutralization validation results of hydrogen peroxide 7% solution

**Neutralizer**	**Sodium thiosulfate in letheen broth**						**MnO_2_ in letheen broth**			**MnO_2_ in D.Wb**			**FeCl_3_ in D.W**		
concentration (w/v)	0.5	0.6	1	1.5	2	3	0.3	0.5	1	0.3	0.5	1	0.5	1
Recovery- test group (CFU/ml)	<10^a^	<10	<10	<10	<10	<10	97	99	95	98	98	97	<10	<10
Recovery- peptone group (CFU/ml)	100	93	91	86	87	78	98	100	94	99	99	96	96	94
Recovery- viability group (CFU/ml)	101	95	96	98	99	98	99	101	95	100	99	97	99	98
Efficacy (%)^b^	<10	<10	<10	<10	<10	<10	99	98	100	99	99	100	<10	<10
Toxicity (%)^c^	99	98	95	88	88	80	99	99	99	99	100	99	97	96

a The results are mean of triplicate counts;

b Ratio of test tube to viability tube count;

C ratio of peptone control group to viability group.

b D.W is stand for distilled water

### Sporicidal tests, Suspension test.

**Fig. 2.**
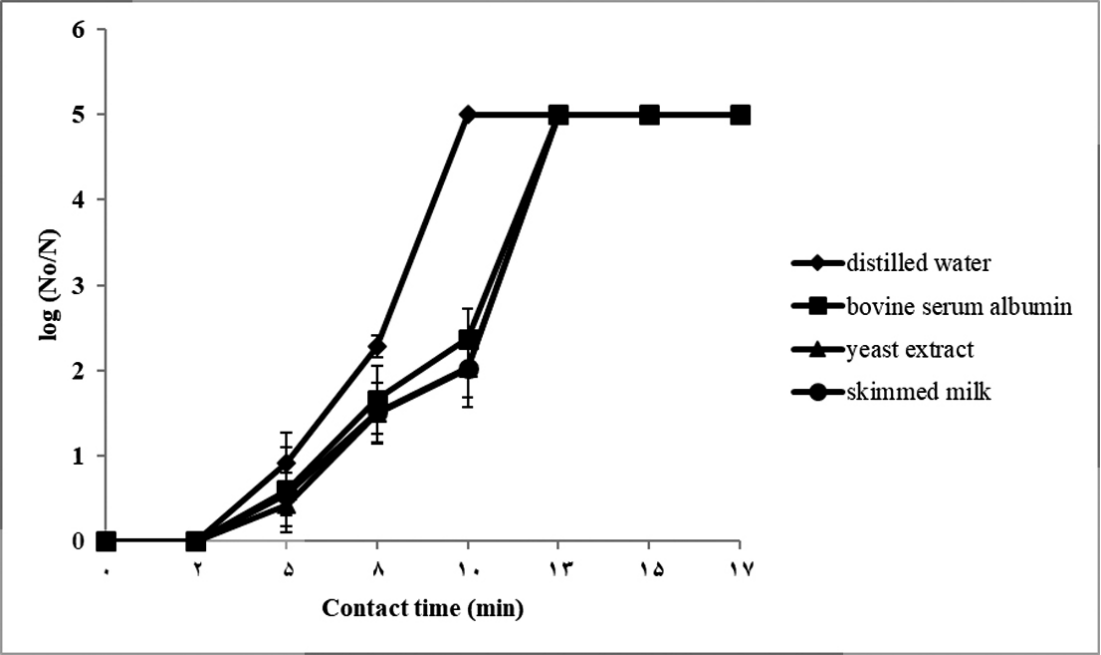
Log reduction ratio of *Bacillus subtilis* spores treated with 7% H_2_O_2_ in distilled water and in the presence of 1 ml interfering substances (0.3% bovine serum albumin, 1% yeast extract, and 1% skimmed milk) according to the suspension test described by BS EN 13704 standard. The initial spore count was 3 × 10^5^ CFU/ml. Results are represented as mean ± S.D.

**Fig. 3.**
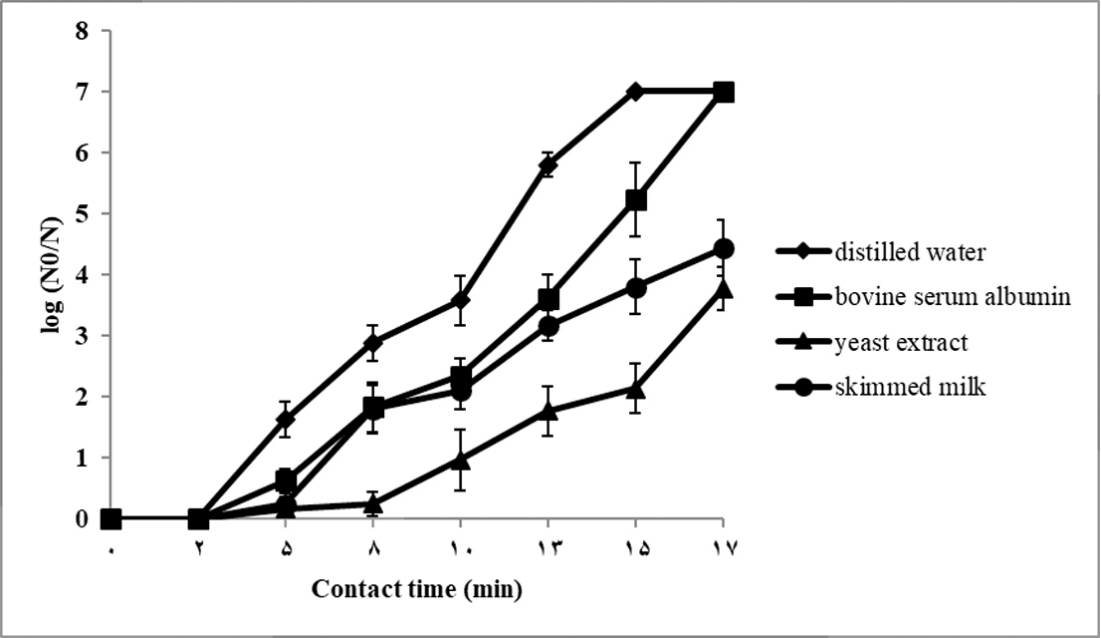
Log reduction ratio of *Bacillus subtilis* spores treated with 7% H_2_O_2_ in distilled water and in the presence of 1 ml interfering substances (0.3% bovine serum albumin, 1% yeast extract and 1% skimmed milk) according to the suspension test described by AFNOR NF 72_230 standard. The initial spore count was 2 × 10^7^ CFU/ml. Results are represented as mean ± S.D.

In both suspension tests, with 3 × 10^5^ and 2 × 10^7^ CFU/ml initial spore counts, the reduction of *B. subtilis* spores were observed after 2 min exposure to 7% H_2_O_2_ (Figs 2 & 3).

As shown in Fig. 2, the log reduction curves for BS EN 13704 suspension tests in the presence of interfering agents were almost overlaid and showed more than 5 log spore reduction at 13 min in the presence of each of three interfering substances. In the presence of interfering agents, the survival counts between 5 and 10 min of exposure were significantly more than that of distilled water (P<0.05).

As shown in Fig. 3, with 2 × 10^7^ CFU/ml initial spore count, spore log-reductions in distilled water were significantly greater than in the presence of interfering agents (P<0.05) at each sampling time. Furthermore, spore log-reductions in the presence of yeast extract or skimmed milk were significantly lower in comparison with bovine serum albumin (P<0.05). The highest spore reduction (more than 7 log) was occurred only with bovine serum albumin (after 17 min) and distilled water (after 15 min).

### Carrier test.

Suspension test is used primarily to assess the disinfectant activity against different microorganisms and to determine the contact time. The next step is the antimicrobial activity evaluation on hard non-porous surfaces e.g. carriers.

In acid-resistance test, all of the test tubes containing inoculated carriers showed visible growth even after 20 min exposure. Therefore, *B. subtilis* spores were qualified to be used as test spores. In the carrier test the no growth (0) was observed after 60 min exposure of *B. subtilis* spores to 7% H_2_O_2_ (Table 2).

**Table 2.** The results of carrier test for 7% H_2_O_2_ according to AOAC MB 15-03 standard. Each carrier was loaded with 5×10^5^ CFU of *Bacillus subtilis* spores.

**Contact time (min)**	**Primary tube (neutralizer)**	**Secondary tube (medium)**					**Result**	**Positive control**	**Control media**		
	
		**(1)**	**(2)**	**(3)**	**(4)**	**(5)**			**(1)**	**(2)**	**(3)**
2	+^a^	+	+	+	+	+	+	+	0^b^	0	0
5	+	+	+	+	+	+	+	+	0	0	0
8	+	+	+	+	+	+	+	+	0	0	0
10	+	+	+	+	+	+	+	+	0	0	0
13	+	+	+	+	+	+	+	+	0	0	0
15	+	+	+	+	+	+	+	+	0	0	0
17	+	+	+	+	+	+	+	+	0	0	0
20	+	+	+	+	+	+	+	+	0	0	0
25	0	+	+	+	+	+	+	+	0	0	0
30	0	+	+	+	+	+	+	+	0	0	0
60	0	0	0	0	0	0	0	+	0	0	0

a growth,

b no growth

In this regard, the required contact time for 5-log reduction of *B. subtilis* spores in distilled water and in the presence of interfering substances are summarized in Table 3. The required time for 5-log spore reduction in dirty conditions and carrier test were significantly higher than clean condition (P<0.05). The contact time with initial count of 3 × 10^5^ CFU/ml was about 9.6 min. Addition of interfering substances increased the exposure time to 12.5 min. When the initial spore concentration of 2 × 10^7^ CFU/ml was used, the contact time was reached to about 11.9 min and interference of bovine serum albumin, skimmed milk, and yeast extract was greater with hydrogen peroxide sporicidal activity. Bovine serum albumin increased the required contact time to about 14.7 min and more that 17 min for skimmed milk and yeast extract. Attachment of bacterial spores to stainless steel hard surfaces in carrier test, increased the exposure time to 60 min.

**Table 3.** Required time for 5-log reduction of *Bacillus subtilis* spores by 7% H_2_O_2_.

**Sporicidal standard test**	**Time (min)**			

	**Distilled water**	**Albumin**	**Skimmed milk**	**Yeast extract**
BS EN 13704 (suspension test with 3 × 10^5^ CFU/ml initial count )	9.64	12.47	12.51	12.51
NF 72-230 (suspension test with 2 × 10^7^ CFU/ml initial count )	11.91	14.71	>17	>17
AOAC MB 15-03 (carrier test with 5 × 10^5^ CFU/ml initial count )	60	NA	NA	NA

NA, not applicable

## DISCUSSION

In this study, the sporicidal activity of 7% H_2_O_2_ against *B. subtilis* spores was tested according to the suspension and carrier tests in both clean and dirty conditions. The suspension test was used primarily to assess the disinfectant activity by using two different initial spore concentrations of 3 × 10^5^ CFU/ml (BS EN 13704 test method) and 2 × 10^7^ CFU/ml (AFNOR NF 72_230 test method). Sagripanti et al. compared the sensitivity of spores of *Bacillus anthracis* and other *Bacillus* spp. to disinfecting agents via carrier test. Their research indicated that sensitivity of the spores of *B. atrophaeus, B. subtilis, B. cereus, B. thuringiensis* and *B. megaterium*, to disinfecting agents is similar to the spores of *B. anthracis* strains. So, a proper conclusions on sporicidal efficacy could be obtained from non-pathogenic spores (20).

In suspension test, the plots of log reduction in spore count with respect to time showed a sigmoid curve with a slower initial reduction in numbers followed by an increasing rate. According to the obtained results, the spore killing rate reduced in the presence of high spore concentration and in the presence of interfering substances. Bovine serum albumin, yeast, and skimmed milk showed similar interfering effects in suspension test with 3 × 10^5^ CFU/ml initial spore count. While, in suspension tests with higher initial spore count (2 × 10^7^ CFU/ml) severity of interfering effects were absolutely intensified and distinct. These outcomes reinforce this hypothesis that initial spore concentration and interfering substances may have the same mechanism of action and work synergistically.

There are two hypotheses to explain the mechanism of interaction among interfering substances and the sporicidal agents. The first one intends the interfering substance as a neutralizer that lead to H_2_O_2_ cytotoxic potency reduction. The second hypothesis says that the presence of interfering substances decreases contact surface between H_2_O_2_ and spores (21). Interfering substances which used in standard tests to simulate natural contaminations are all organic and chemically inactive, so neutralization of H_2_O_2_ by them seems unlikely. Therefore, the second hypothesis seems more probable. The presence of interfering substance and high concentration of spores reduces contact surfaces between H_2_O_2_ and test spores; therefore, the destruction of the permeability barrier of the inner spore membrane by H_2_O_2_ would be delayed (21).

Wesgate and colleagues evaluated different standard test protocols including BS EN 14347, BS EN13704, ASTM E2197-11 and AOAC MB-15-03 on sporicidal efficacy of eight different biocidal agents. They stated that after 5 min of exposure, significant differences in sporicidal activity were found between sporicidal methods but the differences were not significant at 60 min (22). Similarly, we found that by using lower spore concentration, bacterial recoveries in three dirty conditions were relatively the same after 13 min of exposure but the severity of interfering effects were intensified and distinct between 5 to 10 min of exposure. While, bovine serum albumin, yeast, and skimmed milk showed different interfering effects even after 17 min of exposure in suspension test with higher initial spore concentration. In this study, the relatively long time of no-growth observation (60 min) in carrier test is expected by reducing contact surface between H_2_O_2_ and the spores inoculated on the carriers.

## CONCLUSION

The present study was conducted to compare different sporicidal standard test protocols i.e. BS EN 13704 and AFNOR NF 72-230 for suspension tests and AOAC MB-15-04 standard carrier test. Required contact time to reach the effective reduction in spore count is playing an essential role in defining a substance as a sporicidal agent. On the other hand, the presence of proteinaceous and other kind of contaminations in disinfectant usage conditions is unavoidable. Therefore, it is reasonable to use interfering substances or inoculated carriers in accordance with actual conditions of a sporicidal product usage. Also, higher initial spore count in AFNOR NF 72_230 suspension test intensified the interfering effect. So applying suspension test in clean condition to verify the claim of a sporicidal product is strongly discouraged.
